# G3BP2 regulated by the lncRNA LINC01554 facilitates esophageal squamous cell carcinoma metastasis through stabilizing HDGF transcript

**DOI:** 10.1038/s41388-021-02073-0

**Published:** 2021-11-15

**Authors:** Yinli Zheng, Jinjun Wu, Ru Deng, Censhan Lin, Yuhua Huang, Xia Yang, Chunhua Wang, Mingming Yang, Yangfan He, Jiabin Lu, Xiaodong Su, Qian Yan, Yinghui Zhu, Xinyuan Guan, Yan Li, Jingping Yun

**Affiliations:** 1 Sun Yat-sen University Cancer Center, State Key Laboratory of Oncology in South China, Collaborative Innovation Center for Cancer Medicine, Guangzhou, 510060 P. R. China; 2 Department of Pathology, Sun Yat-sen University Cancer Center, Guangzhou, 510060 PR China; 3 Joint Laboratory for Translational Cancer Research of Chinese Medicine of the Ministry of Education of the People’s Republic of China, International Institute for Translational Chinese Medicine, Guangzhou University of Chinese Medicine, Guangzhou, 510006 PR China; 4 Department of Thoracic Surgery, Sun Yat-sen University Cancer Center, Guangzhou, 510060 PR China; 5 Department of Colorectal Surgery, Guangdong Institute of Gastroenterology, Guangdong Provincial Key Laboratory of Colorectal and Pelvic Floor Diseases, The Sixth Affiliated Hospital, Sun Yat-sen University, Guangzhou, 510000 PR China; 6 Department of Clinical Oncology, State Key Laboratory for Liver Research, The University of Hong Kong, Hong Kong, 852 PR China; 7 Department of Clinical Oncology, The University of Hong Kong-Shenzhen Hospital, Shenzhen, 518053 PR China

**Keywords:** Metastasis, Cell migration, Prognostic markers

## Abstract

Metastasis is the leading cause of death of patients with esophageal squamous cell carcinoma (ESCC). Although an increasing number of studies have demonstrated the involvement of G3BP2 in several human cancers, how G3BP2 interacts with long noncoding RNAs and regulates mRNA transcripts in mediating ESCC metastasis remains unclear. In this study, we uncovered that G3BP2 was upregulated in ESCC. Further analysis revealed that upregulation of G3BP2 was significantly correlated with lymph node metastasis, depth of tumor invasion and unfavorable outcomes in ESCC patients. Both in vitro and in vivo functional assays demonstrated that G3BP2 dramatically enhanced ESCC cell migration and invasion. Mechanistically, LINC01554 maintained the high G3BP2 expression in ESCC by protecting G3BP2 from degradation through ubiquitination and the interaction domains within LINC01554 and G3BP2 were identified. In addition, RNA-seq revealed that HDGF was regulated by G3BP2. G3BP2 bound to HDGF mRNA transcript to stabilize its expression. Ectopic expression of HDGF effectively abolished the G3BP2 depletion-mediated inhibitory effect on tumor cell migration. Intriguingly, introduction of compound C108 which can inhibit G3BP2 remarkedly suppressed ESCC cell metastasis in vitro and in vivo. Collectively, this study describes a newly discovered regulatory axis, LINC01554/G3BP2/HDGF, that facilitates ESCC metastasis and will provide novel therapeutic strategies for ESCC.

## Introduction

Esophageal carcinoma (EC) is an exceedingly aggressive cancer with a high mortality rate and poor prognosis worldwide [1], and squamous cell carcinoma is the major pathological type of EC [2]. Clinically, metastasis is the ultimate challenge for effectively treating ESCC. The lymph nodes and lung are the most frequent metastatic organs. To date, although several whole-exon sequencing studies have identified universally mutated genes that illustrate the genetic alterations in patients with ESCC [3–5] and lots of research on ESCC metastasis have been carried out [6, 7], the precise molecular mechanisms and complexity of ESCC metastasis are still not fully understood. Thus, discovering the mechanisms of the dissemination ESCC cells will be valuable for the development of novel therapeutic approaches for ESCC.

Ras-GTPase-activating protein (SH3 domain)-binding protein 2 (G3BP2) is located on the long arm of chromosome 4 (4q21), which has been reported to harbor genetic variant risk factors for ESCC in the Chinese population [8]. G3BP2 is mainly distributed in the cytoplasm and participates in the formation of stress granules [9], cell differentiation, proliferation, and signal transduction [10]. Accumulating evidence has demonstrated that aberrant expression of G3BP2 contributes to cancer initiation and progression, such as high expression of G3BP2 increasing cell stemness, metastasis and chemoresistance in breast cancer [11–13], upregulation of G3BP2 promoting cell growth and survival in prostate cancer [14], and bladder cancer [15]. In addition, G3BP2 attenuates the effectiveness of radiation in the treatment of oral cancer [16]. However, the function and mechanism of G3BP2 in ESCC metastasis have yet to be elucidated.

G3BP2 belongs to the G3BP protein family, which has four distinct motifs in its protein structure, including a nuclear transport factor 2-like domain, acidic and proline-rich regions, an RNA recognition motif (RRM) and an arginine and glycine rich box (RGG box). Among the motifs, the RRM domain is the most common specific basic domain of RNA binding proteins (RBPs) [17]. Mounting evidence has revealed that RBPs engage in posttranscriptional regulation by modulating RNA splicing, polyadenylation, mRNA stability, mRNA localization, and translation through interacting with coding or noncoding RNAs [18]. Therefore, RBPs are critical for maintaining the homeostasis of gene expression. Abnormal expression of RBPs disrupts gene expression homeostasis and causes human diseases, especially cancers. As RBPs, G3BPs have been reported to participate in the regulation of gene expression by modulating mRNA stability and translation under the stimulation of environmental stresses in several diseases, including cancer progression and virus survival [10]. Nevertheless, how G3BP2 interacts with mRNAs in ESCC remain to be explored.

Long noncoding RNAs (lncRNAs), transcribed RNAs of more than 200 nucleotides in length, engage in diverse biological processes involved in tumor initiation, growth and metastasis at the epigenetic, transcriptional, posttranscriptional, and translational levels [19]. LncRNAs has been reported to most frequently modulate the expression, conformation, and localization of target proteins [20]. In our study, both the RNA–protein interaction prediction platforms catRAPID (http://service.tartaglialab.com) and RPI-seq (http://pridb.gdcb.iastate.edu) suggest that G3BP2 may interact with the lncRNA LINC01554. LINC01554 is a long intergenic noncoding RNA and is mainly localized in the cytoplasm. It has been demonstrated to attenuate cell growth by inhibiting glycolytic effects [21] and acting as a miRNA sponge to interfere with NGFR expression in hepatocellular carcinoma (HCC) [22]. However, the role of LINC01554 in ESCC and how LINC01554 modulates G3BP2 have not been fully illustrated.

In this study, our data revealed that G3BP2 was frequently upregulated in ESCC tissues and predicted unfavorable patient outcomes. Mechanistically, LINC01554 maintained the high expression of G3BP2 by inhibiting ubiquitin mediated proteasome degradation of G3BP2. RNA-seq further identified hepatoma-derived growth factor (HDGF) as the objective mRNA that regulated by G3BP2. G3BP2 stabilized HDGF mRNA transcript to maintain its high expression level and then increased ESCC metastasis. Notably, the introduction of compound C108, a small molecule that inhibits G3BP2, markedly attenuated ESCC cell metastasis in vitro and in vivo. In conclusion, our findings describe a new regulatory signaling axis, LINC01554/G3BP2/HDGF, that facilitates ESCC metastasis, and highlight that G3BP2 could be a promising therapeutic target for treatment of ESCC.

## Results

### Upregulation of G3BP2 is correlated with unfavorable outcomes in ESCC patients

**Fig. 1:**
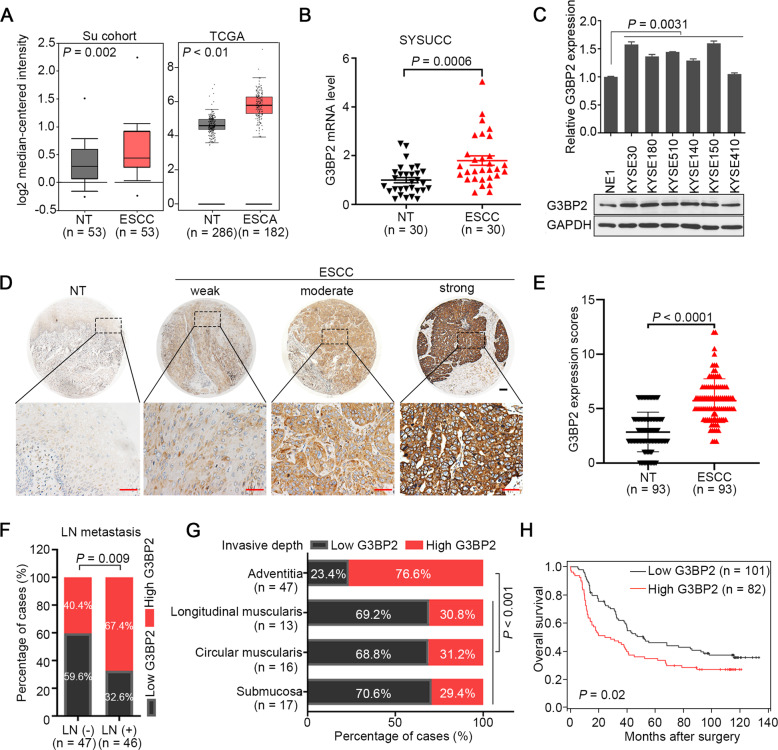
Upregulation of G3BP2 is correlated with unfavorable outcome in ESCC patients. **A** Big data mining from Oncomine (https://www.oncomine.org) and TCGA (https://www.cancer.gov) showed that G3BP2 was upregulated in ESCC. **B** mRNA expression of G3BP2 was examined by RT-qPCR using SYSUCC samples. GAPDH served as an internal control. **C** The protein expression of G3BP2 in immortalized esophageal epithelium cells (NE1) and ESCC cell lines. GAPDH acted as a loading control. **D** Representative images of G3BP2 staining in nontumor tissue and ESCC tissue microarray by immunohistochemistry analysis. Scale bar, up: 100 μm, down: 50 μm. **E** IHC score of G3BP2 staining in ESCCs and corresponding nontumor samples (n = 93). **F** and **G** Clinicopathologic correlation analyses of G3BP2 expression in ESCC. **H** Correlation between G3BP2 expression and overall survival was analyzed in the SYSUCC cohort by Kaplan-Meier curve analysis.

To investigate the expression status of G3BP2 in ESCC, we first performed big data mining from Oncomine (https://www.oncomine.org) and TCGA databases (https://www.cancer.gov) Both databases indicated that G3BP2 was highly expressed in ESCC (Fig. 1A), which was further confirmed in the SYSUCC cohort at both the mRNA (Fig. 1B) and protein levels (Supplementary Fig. 1). Elevated expression of G3BP2 was also detected by Western blot analysis in ESCC cells (Fig. 1C). To investigate the clinical significance of upregulated G3BP2 expression in ESCC, a tissue microarray containing 183 primary ESCCs and 93 paired nontumor tissues was studied by IHC staining (Fig. 1D). The result indicated the high expression of G3BP2 in ESCC tissues (*P* < 0.0001, Fig. 1E). Furthermore, high G3BP2 expression was significantly associated with lymph node metastasis (*P* = 0.009, Fig. 1F) and the depth of tumor invasion (*P* < 0.001, Fig. 1G, Supplementary Table 1). Kaplan-Meier analysis demonstrated that high G3BP2 expression contributed to poorer overall survival of ESCC patients (*P* = 0.02, Fig. 1H). All these data illustrate the robustness of the correlation between high G3BP2 expression levels and ESCC progression.

### LINC01554 protects G3BP2 from ubiquitin proteasome degradation

**Fig. 2:**
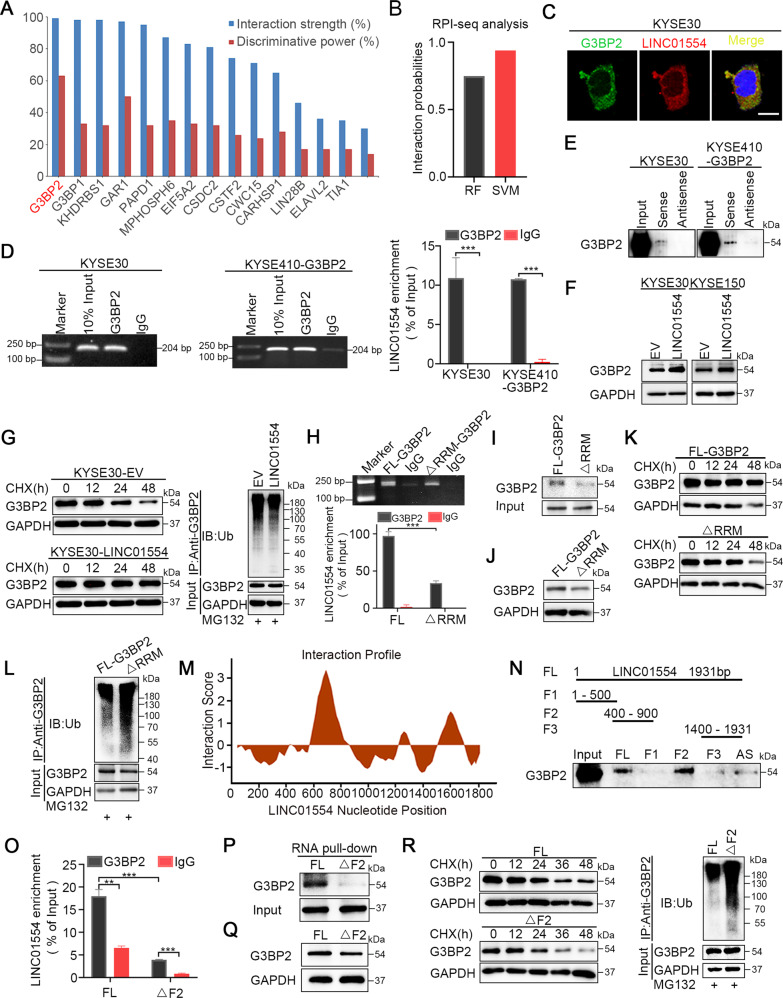
LINC01554 protects G3BP2 from ubiquitin-proteasome degradation. **A** catRAPID (http://service.tartaglialab.com) suggested the interaction between G3BP2 and LINC01554. **B** RPI-seq platforms (http://pridb.gdcb.iastate.edu) strongly recommended the possibility of interaction between G3BP2 and LINC01554. **C** Colocalization of G3BP2 and LINC01554 was detected by FISH-IF. Scale bar, 20 μm. RIP (**D**) and RNA pull-down (**E**) assays showed the interaction of G3BP2 with LINC01554. IgG antibody served as a control for RIP assay. **F** Western blot analysis revealed that the protein expression of G3BP2 was upregulated by LINC01554 in LINC01554-transfected KYSE30 and KYSE150 cells. **G** Cycloheximide chase assay showed that the half-life of the G3BP2 protein in LINC01554-transfected KYSE30 cells was longer than that in empty vector cells (left). In vitro ubiquitination assay showed that the ubiquitination level of the G3BP2 protein was abolished in LINC01554-transfected KYSE30 cells (right). RIP (**H**) and RNA pull-down (**I**) assays showed that G3BP2 did not bind to LINC01554 after deletion of the RRM domain in G3BP2. IgG antibody served as a control for the RIP assay. FL-G3BP2, full-length G3BP2; ΔRRM-G3BP2, truncation of the RRM domain in G3BP2. **J** Western blot showed that the G3BP2 protein level was decreased in KYSE30 cells with truncation of the RRM domain. **K** Cycloheximide chase assay showed that the half-life of the G3BP2 protein was shorter in KYSE30 cells with truncation of the RRM domain than in cells with full-length G3BP2. **L** In vitro ubiquitination assay showed that the ubiquitination level of the G3BP2 protein was dramatically increased in KYSE30 cells with truncation of the RRM domain. **M** catRAPID platform (http://service.tartaglialab.com) was applied to predict the specific binding sequence of LINC01554 with G3BP2 protein. **N** Top, schematic structures showing a summary of in vitro transcribed, biotinylated full-length, truncated or antisense LINC01554 probes. Bottom, immunoblot analysis with anti-G3BP2 in KYSE30 cells followed by RNA pull-down assay with full-length, truncated or antisense LINC01554 probes. FL, full length (1–1931 bp); F1, fragment 1 (1–500 bp); F2, fragment 2 (400–900 bp); F3, fragment 3 (1400–1931 bp); AS, antisense. RIP (**O**) and RNA pull-down (**P**) assays showed that the interaction of G3BP2 and LINC01554 was weakened in KYSE30 cells transfected with truncated fragment 2 in LINC01554. IgG antibody served as a control for RIP assay. **Q** Western blot showed that the G3BP2 protein level declined in KYSE30 cells transfected with truncated fragment 2 within LINC01554. **R** Cycloheximide chase assay showed that the half-life of the G3BP2 protein with truncated fragment 2 in LINC01554-transfected KYSE30 cells was shorter than that in full-length transfected cells (left). In vitro ubiquitination assay showed that the ubiquitination level of the G3BP2 protein was elevated in KYSE30 cells transfected with LINC01554 with fragment 2 truncated (right). The data represent the mean ± SD from three biological replicates. **P < 0.01, ***P < 0.001.

According to the structural characteristics of G3BP2, which harbors an RNA recognition motif, we asked whether it interacted with noncoding RNAs. Both the catRAPID and RPI-seq platforms strongly suggested the possibility of an interaction between G3BP2 and LINC01554 (Fig. 2A, B). Then, RNA FISH-IF, RIP, and RNA pull-down assays were conducted to validate the prediction results. The results of the RNA FISH-IF assay showed that G3BP2 and LINC01554 colocalized in the cytoplasm (Fig. 2C). RIP assay presented that G3BP2 could specifically precipitate LINC01554 (Fig. 2D). RNA pull-down assays further demonstrated that the LINC01554 probe, but not its antisense probe, significantly pull down G3BP2 protein (Fig. 2E). In light of the accumulating evidence showing that lncRNAs may modulate their binding objects [19, 20], we examined whether LINC01554 regulated G3BP2 expression. Both G3BP2 RNA and protein were detected in LINC01554-transfected KYSE30 and KYSE150 cells, and we observed that G3BP2 was increased at protein level but not RNA level after transfection of LINC01554 (Fig. 2F and Supplementary Fig. 2A, B). And silencing LINC01554 declined G3BP2 protein expression (Supplementary Fig. 2C, D). Next, we explored how LINC01554 increased G3BP2 protein expression. Since recent studies have illustrated that lncRNAs regulated the protein levels of their target genes by interfering with the protein stability [23, 24], a cycloheximide chase assay was carried out to verify our hypothesis. The results showed that the half-life of the G3BP2 protein was obviously longer in LINC01554-transfected cells (Fig. 2G, left panel). To examine whether G3BP2 degradation occurred via the ubiquitin-mediated proteasome system, an in vitro ubiquitination assay was performed, and it was found that LINC01554 decreased the level of G3BP2 ubiquitination (Fig. 2G, right panel). Moreover, the reduction of G3BP2 in LINC01554 knockdown cells was rescued when treated with MG132 (Supplementary Fig. 2E). Therefore, these data show that LINC01554 interacts with G3BP2 and protects it from protein degradation mediated by the ubiquitin-proteasome system.

To confirm that the RRM domain of G3BP2 directed the interaction with LINC01554, we established G3BP2 mutants with deletion of the RRM domain. The results of the RIP (Fig. 2H) and RNA pull-down assays (Fig. 2I) demonstrated that the RRM domain of G3BP2 indeed bound to LINC01554. However, we observed that in spite of deletion of RRM domain, there was still about 40% of LINC01554 bound to G3BP2. To eliminate the possibility of other domains in G3BP2 interacted with LINC01554, RIP assays were conducted in cells transfected with a series of truncation mutant constructs. The result showed that only removing RRM domain impaired the interaction between G3BP2 and LINC01554 (Supplementary Fig. 2F). After elimination of the RRM domain, protein expression of G3BP2 was decreased due to enhanced protein degradation through ubiquitination (Fig. 2J–L). Next, we used catRAPID platform to predict the specific binding sequences within LINC01554 that was essential for its interaction with G3BP2. The higher absolute value of interaction score indicated the greater possibility of the interaction. As Fig. 2M showed, nucleotide sequences of 1–600 bp presented low interaction score, while that of 600–800 bp displayed the highest interaction score. Sequences of 1400–1931 bp also presented the relatively high interaction score. According to the above result and considering the length of secondary structure of LINC01554, the actual binding sequences of LINC01554 with G3BP2 might range from 400–900 bp. Thereafter, we constructed and biotinylated three truncated fragments of LINC01554 (fragment 1: 1–500 bp, fragment 2: 400–900 bp, fragment 3: 1400–1931 bp) for use in an RNA pull-down assay with KYSE30 cell lysates, and found that fragment 2 of LINC01554 indeed pulled down G3BP2, but not other fragments (Fig. 2N). To further examine the indispensable function of fragment 2 in LINC01554 interacting with G3BP2, a LINC01554 mutant with a fragment 2 deletion was constructed. The results of the RIP and RNA pull-down assays revealed the indispensable role of fragment 2 in mediating the interaction of LINC01554 with G3BP2 (Fig. 2O, P). In addition, G3BP2 protein expression was decreased after fragment 2 was removed from LINC01554 owing to increased ubiquitin-mediated degradation of the G3BP2 protein (Fig. 2Q, R and Supplementary Fig. 2G). Collectively, these findings show that LINC01554 binds to the RRM domain of G3BP2 mainly through the nucleotide domain ranging from 400 to 900 bp.

### G3BP2 enhances ESCC cell metastasis in vitro and in vivo

**Fig. 3:**
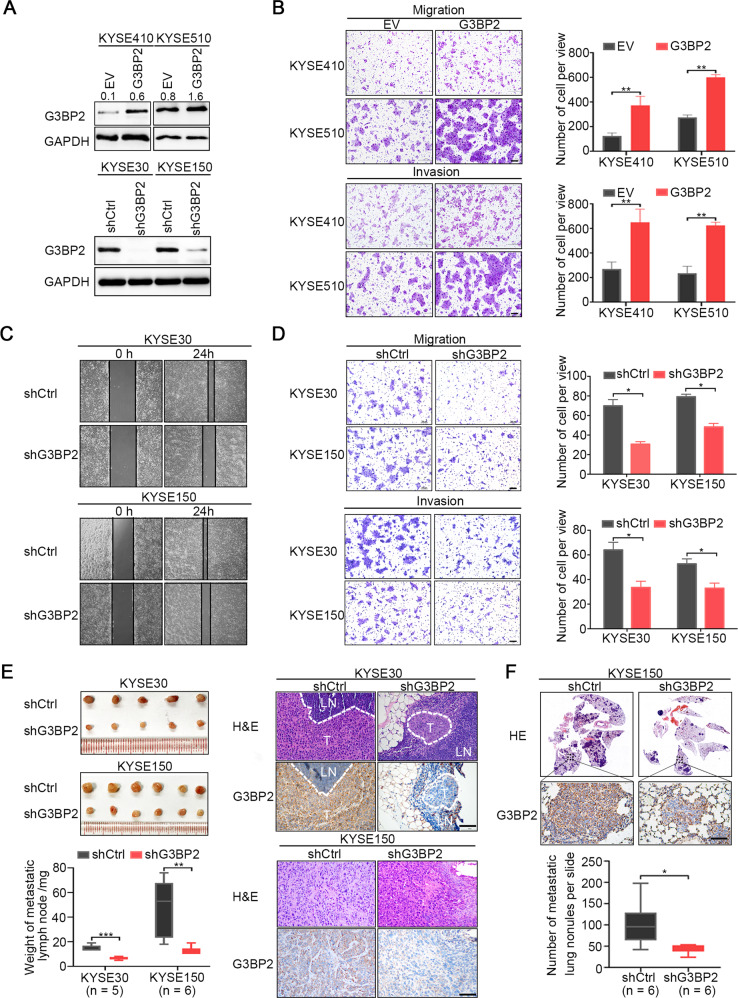
G3BP2 enhances ESCC cell metastasis in vitro and in vivo. **A** The ectopic expression and knockdown efficiency of G3BP2 were detected by Western blot. **B** Transwell assays revealed that ectopic expression of G3BP2 enhances cell migration and invasion in KYSE410 and KYSE510 cells. Scale bar, 50 μm. Data are presented as the mean ± SD of three biological replicates. **C** Wound healing assay showed that silencing G3BP2 significantly impaired cell migration in KYSE30 and KYSE150 cells. Representative images were captured 24 h after scratching. **D** Transwell assays showed that knocking down G3BP2 attenuated the migration and invasion capability of KYSE30 and KYSE150 cells. Scale bar, 50 μm. The data are expressed as the mean ± SD of three biological replicates. **E** An in vivo inguinal lymph node metastatic model showed smaller size and lighter weight of lymph nodes with weak G3BP2 staining in the G3BP2-knockdown group compared with metastatic lymph nodes in the control group (*n* = 5 mice/group of KYSE30 cells, *n* = 6 mice/group of KYSE150 cells). Scale bar, 50 μm. **F** In vivo lung metastatic model via tail venous injection presented fewer metastatic nodules under a microscope and weaker G3BP2 staining by IHC in the G3BP2 silenced group (*n* = 6 mice/group). Black arrows indicate metastatic nodules formed in the lung. Scale bar, 50 μm. The data represent the mean ± SD. *P < 0.05, **P < 0.01.

Given the clinical significance and mechanism of elevated G3BP2 expression in ESCC, we next explored the roles of G3BP2 in ESCC progression. Above all, stable cell lines with ectopic expression or knockdown of G3BP2 were established in low endogenous G3BP2 expression cells (KYSE410 and KYSE510 cells) and high endogenous G3BP2 expression cells (KYSE30 and KYSE150 cells), respectively. The overexpressed and knockdown efficiency was examined by Western blot (Fig. 3A). Ectopic expression of G3BP2 in KYSE410 and KYSE510 cells dramatically increased ESCC cell migration and invasion (Fig. 3B), while knocking down G3BP2 in KYSE30 and KYSE150 cells remarkedly attenuated cell metastatic ability (Fig. 3C, D), but did not affect cell growth (Supplementary Fig. 3). We further evaluated the function of G3BP2 in vivo using both lymph node and lung metastatic nude mouse models. Four weeks after injection of G3BP2-knockdown cells and corresponding control cells, the mice were sacrificed. The inguinal lymph nodes or lungs were then isolated for observation. In lymph node metastasis model mice, smaller and lighter lymph nodes with lower expression of G3BP2 were observed in the knockdown group (Fig. 3E). In lung metastatic model mice, we also found less metastatic nodules and lower G3BP2 expression under microscope in lung tissues isolated from the knockdown group than from the control group (Fig. 3F). Taken together, these results imply the important role of G3BP2 in promoting ESCC metastasis, which is consistent with its clinical significance.

### G3BP2 stabilizes HDGF mRNA transcript

**Fig. 4:**
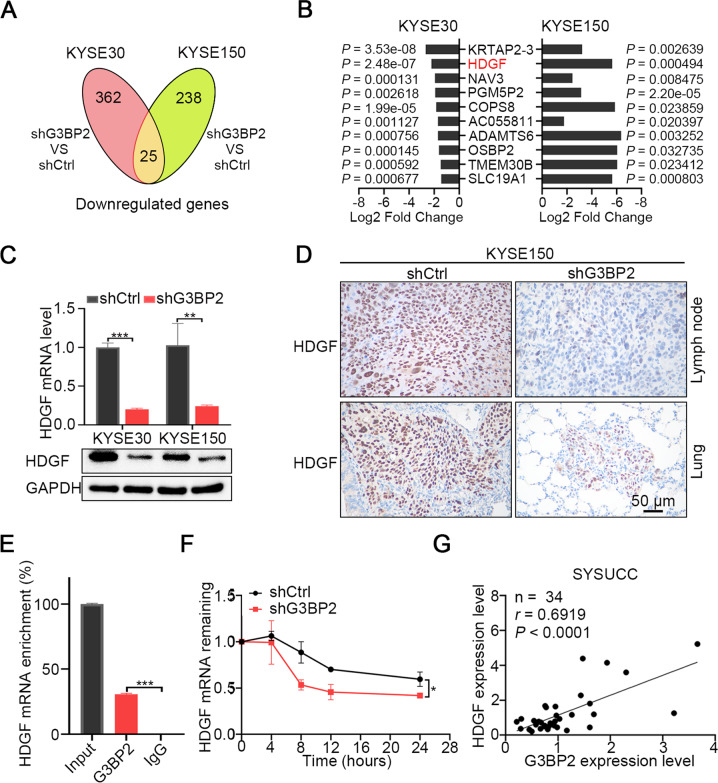
G3BP2 stabilizes HDGF mRNA transcript. **A** Venn diagram filtering 25 shared genes among the downregulated differential genes in KYSE30 and KYSE150 cells with G3BP2 knocked down. **B** Fold changes and *P* values of the top ten shared differential genes in KYSE30 and KYSE150 cells with G3BP2 silencing were displayed. **C** mRNA and protein expression of HDGF was determined by RT-qPCR and Western blot, respectively, in KYSE30 and KYSE150 cells with G3BP2 silencing. GAPDH was an endogenous control. The data represent the mean ± SD of three biological replicates. ***P < 0.001. **D** IHC staining of HDGF in tissues of metastatic tumors formed in the lymph node (upper) and lung (lower) through footpad or tail venous injection of KYSE150 cells with G3BP2 knocked down. Scale bar, 50 μm. **E** RIP assay revealed the binding of G3BP2 and HDGF mRNA transcript. IgG antibody served as a control. The data represent the mean ± SD from triplicate experiments. ***P < 0.001. **F** G3BP2 knockdown in KYSE150 cells significantly shortened the half-life of HDGF mRNA transcript. The data represent the mean ± SD from three independent experiments. *P < 0.05. **G** G3BP2 RNA expression showing a positive correlation with HDGF RNA expression in 34 ESCC samples collected from SYSUCC.

To identify the mRNA transcripts regulated by G3BP2 involved in inducing ESCC metastasis, we carried out RNA-seq on G3BP2-knockdown KYSE30 and KYSE150 cells. Based on the filtering criteria of *P* < 0.05 and log2 ^(fold change)^ > 1, 387 and 263 differentially downregulated genes were identified in KYSE30 cells and KYSE150 cells, respectively, among which 25 genes were shared in both cell lines (Fig. 4A). By combining the fold change and significant *P* value results, we found that HDGF presented a large fold change with a very significant *P* value in both KYSE30 and KYSE150 cells among the top 10 differentially downregulated genes (Fig. 4B). In particular, accumulating studies have shown that HDGF plays an important role in driving tumor cell metastasis [25].

To validate the RNA-seq result, we detected both the RNA and protein levels of HDGF in stable G3BP2-knockdown cell lines and found that they were significantly decreased compared with those in control cells (Fig. 4C). IHC staining analysis also showed the low expression of HDGF in metastatic lymph nodes and lung tissue derived from G3BP2-knockdown KYSE150 cells (Fig. 4D). We next sought to figure out how G3BP2 modulated HDGF expression. G3BP2 belongs to class of RNA binding proteins and has been reported to stabilize the SART3 mRNA transcript through its RNA binding domain [11]. Based on that, we deduced G3BP2 regulated HDGF expression by similar mechanisms. A RIP assay was performed to verify our speculation and found that G3BP2 is indeed bound to HDGF mRNA transcript (Fig. 4E). Next, the mRNA half-life assay revealed that silencing G3BP2 led to a significant reduction of the HDGF mRNA stability (Fig. 4F). To examine whether G3BP2 interacted with HDGF through RRM domain, RIP assay and RNA stability assay were further performed in RRM domain truncated- and empty vector- cells. We found that HDGF mRNA enrichment and RNA stability were significant decreased in cells with the deleted RRM domain (Supplementary Fig. 4A, B). Finally, to clarify the correlation between G3BP2 and HDGF expression in ESCC, we first explore the expression status of HDGF in ESCC using public data (http://ualcan.path.uab.edu) and the result revealed that HDGF was upregulated in ESCC (Supplementary Fig. 4C), which was consistent with the expression status of G3BP2 in ESCC. Then, we detected the RNA expression levels of G3BP2 and HDGF in an SYSUCC cohort of 34 ESCC samples, and found that the expression of G3BP2 and HDGF was positively correlated (*P* < 0.0001, Fig. 4G). These findings suggest that G3BP2 modulates HDGF expression by stabilizing its mRNA transcript.

### The LINC01554/G3BP2/HDGF signaling axis facilitates ESCC cell metastasis

**Fig. 5:**
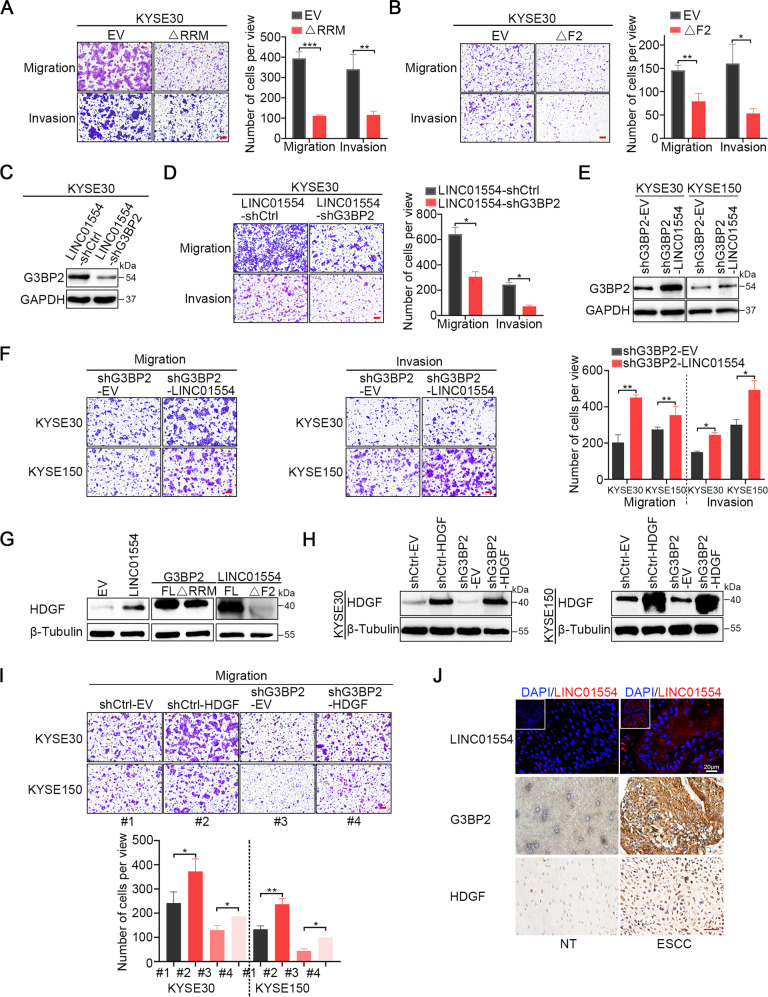
LINC01554/G3BP2/HDGF signaling axis facilitates ESCC cell metastasis. **A** Transwell assays revealed that migration and invasion ability was abrogated in KYSE30 cells with elimination of the RRM domain compared with cells with empty vector. Scale bar, 50 μm. **B** Transwell assays revealed that cell migration and invasion capability were compromised in KYSE30 cells with fragment 2 truncated compared with cells with empty vector. Scale bar, 50 μm. **C** Western blot showed that the effect of LINC01554 upregulation on G3BP2 protein expression was abolished by G3BP2 shRNA in KYSE30 cells. **D** The stimulation of cell migration and invasion by ectopic expression of LINC01554 in KYSE30 cells was reversed by applying shRNA of G3BP2. Left, representative images of Transwell assays; scale bar, 50 μm. Right, statistical analysis of Transwell assays. **E** Immunoblot analysis showed that LINC01554 rescued the protein expression of G3BP2 in KYSE30 and KYSE150 cells with G3BP2 silencing. **F** Decreased cell migration and invasion of G3BP2 knockdown-KYSE30 and -KYSE150 cells was rescued by overexpression of LINC01554. Left and middle, representative images of the Transwell assays; scale bar, 50 μm. Right, statistical analysis of the Transwell assays. **G** Immunoblot analysis presenting the protein expression of HDGF in KYSE30 cells transfected with LINC01554, RRM-truncated G3BP2, fragment 2-truncated LINC01554, and matched control constructs. β-Tubulin served as an internal control. FL, full length; F2, fragment 2; ΔRRM, truncation of RRM. **H** Western blot analysis showed that decreased protein expression of HDGF induced by silencing G3BP2 was rescued by ectopic expression of HDGF in KYSE30 and KYSE150 cells. β-Tubulin served as an internal control. **I** Inhibited migration of KYSE30 and KYSE150 cells with G3BP2 knocked down was rescued by the overexpression of HDGF. Upper, representative images of Transwell migration assays, scale bar, 100 μm. Lower, statistical analysis of Transwell migration assays. **J** Representative images of LINC01554, G3BP2 and HDGF expressions in serial section of clinical samples. Scale bar, 20 μm for RAN-FISH assay, 50 μm for IHC staining. The data represent the mean ± SD from triplicate experiments. *P < 0.05, **P < 0.01, ***P < 0.001.

Given that LINC01554 controlled the protein stability of G3BP2 and HDGF mRNA transcript was regulated by G3BP2, we then investigated whether the LINC01554/G3BP2/HDGF regulatory signaling axis played a critical role in driving ESCC metastasis. First, the effect of the interaction between G3BP2 and LINC01554 in ESCC metastasis was validated by migration and invasion transwell assays. The results showed that elimination of either the RRM domain in G3BP2 or fragment 2 within LINC01554 in KYSE30 cells significantly attenuated cell migration and invasion (Fig. 5A, B). However, the expression status and function of LINC01554 in ESCC were still confusing. The RT-qPCR results showed that LINC01554 was highly expressed in ESCC (Supplementary Fig. 5A). Transwell assay presented that overexpressing LINC01554 increased ESCC cell migratory and invasive abilities (Supplementary Fig. 5B), while silencing LINC01554 dramatically attenuated the migration of ESCC cells (Supplementary Fig. 5C). To confirm that G3BP2 mediated LINC01554-facilitated ESCC cell metastasis, G3BP2 was then silenced in LINC01554-transfected KYSE30 cells. we found that G3BP2 protein expression was markedly decreased (supplementary Fig. 5D, Fig. 5C) and that cell metastasis was abolished (Supplementary Fig. 5E, Fig. 5D). Besides, overexpression of G3BP2 in LINC01554-knockdown cells significantly eliminated the metastasis-suppressive function upon LINC01554 silencing (Supplementary Fig 5F). Moreover, induced expression of LINC01554 reversed G3BP2 expression (Supplementary Fig. 5G, Fig. 5E) and functionally rescued the decrease in cell migration and invasion upon G3BP2 silencing (Supplementary Fig. 5H, Fig. 5F). Considering that HDGF was regulated by G3BP2, we detected the expression of HDGF in KYSE30 cells after transfection of LINC01554 or release of the interaction between LINC01554 and G3BP2. The results showed that HDGF expression was elevated after ectopically expressing LINC01554 but decreased when the domain responsible for the interaction between LINC01554 and G3BP2 was deleted (Fig. 5G). To elucidate the pivotal role of HDGF in the G3BP2 mediated ESCC metastasis, we expressed HDGF in G3BP2-knockdown cells and found that the protein expression of HDGF was rescued (Fig. 5H). Transwell migration assay showed that overexpressing HDGF promoted ESCC cell migration and effectively rescued the reduction in cell migration induced by silencing G3BP2 (Fig. 5I). Conversely, knocking down HDGF remarkably inhibited cell migration and abolished the metastasis-promoting role of G3BP2 (Supplementary Fig. 5I). Eventually, we detected the expression of LINC01554 with RNA FISH and expression of G3BP2 as well as HDGF with IHC staining in three pairs of ESCC and nontumor samples, and observed that the expression of LINC01554, G3BP2, and HDGF showed the same pattern. High expression of LINC01554 was observed with increased G3BP2 and HDGF expression in ESCC, while low expression of LINC01554 was observed with decreased expression of G3BP2 and HDGF in corresponding nontumor tissues (Fig. 5J). Taken together, our results indicate the essential role of the LINC01554/G3BP2/HDGF signaling axis in facilitating ESCC metastasis.

### A G3BP2 inhibitor compound C108 effectively abrogates ESCC cell metastasis in vitro and in vivo

**Fig. 6:**
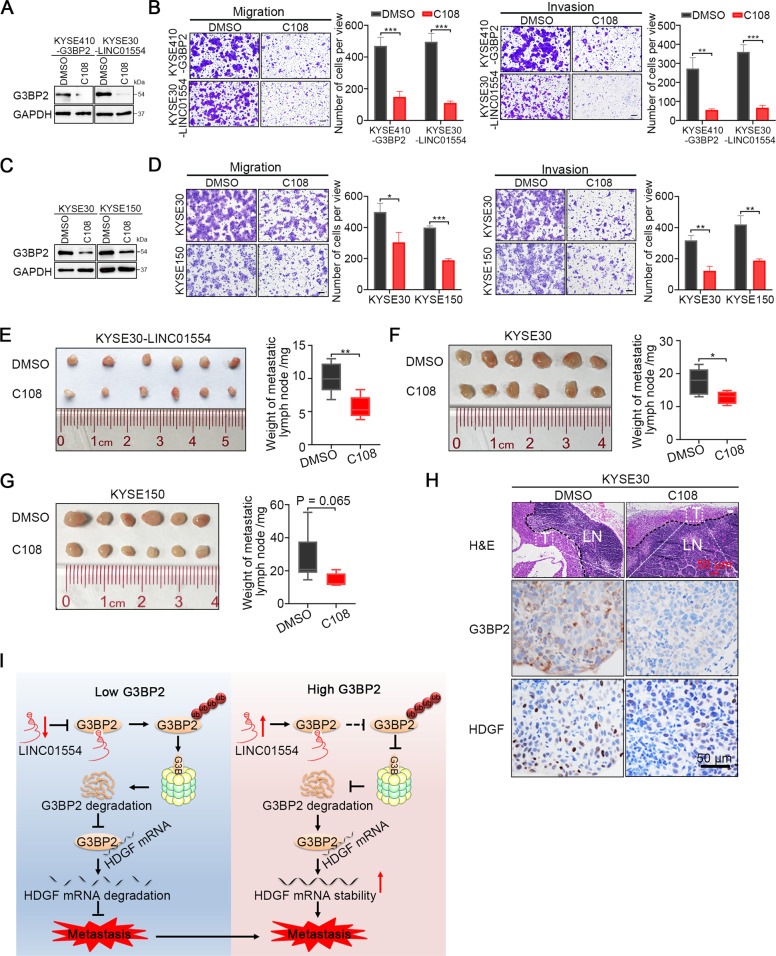
G3BP2 inhibitor compound C108 effectively abrogates ESCC cell metastasis in vitro and in vivo. **A** Western blot analysis showed that the compound C108 significantly decreased the protein expression of G3BP2 in G3BP2 overexpressing KYSE410 cells and LINC01554 transfected KYSE30 cells compared with cells treated with DMSO. **B** Compound C108 dramatically abrogated migration and invasion in G3BP2 overexpressing KYSE410 cells compared with cells treated with DMSO. Scale bar, 50 μm. The data represent the mean ± SD of three independent experiments. **P < 0.01, ***P < 0.001. **C** Western blot analysis showed that the compound C108 obviously decreased G3BP2 protein expression in KYSE30 and KYSE150 cells compared with cells treated with DMSO. **D** Compound C108 markedly attenuated the migration and invasion ability of KYSE30 and KYSE150 cells compared with cells treated with DMSO. Scale bar, 50 μm. The data are expressed as the mean ± SD of three biological replicates. *P < 0.05, **P < 0.01, ***P < 0.001. Metastatic inguinal lymph nodes derived from LINC01554 transfected KYSE30 cells (**E**), KYSE30 cells (**F**), and KYSE150 cells (**G**) respectively, and corresponding graphs showing the weight of lymph nodes at the end of treatment with compound C108 and DMSO. *P < 0.05, **P < 0.01. The data represent the mean ± SD. **H** H&E, G3BP2, and HDGF staining in serial section of metastatic lymph nodes isolated from KYSE30 treated with compound C108 and DMSO. Scale bar, 50 μm. **I** A schematic diagram of the LINC01554/G3BP2/HDGF regulatory signaling axis that facilitates ESCC metastasis.

Compound C108, a small molecule that inhibits several proteins including G3BP2, was developed by Nisha Gupta et al. in 2017. It was reported to block the RRM domain and then impaired the stability of SART3 mRNA, subsequently leading to tumor suppression in breast cancer [11]. To test whether compound C108 could abrogate the interaction between G3BP2 and LINC01554 and potentially be used to block ESCC metastasis induced by G3BP2, we first treated G3BP2-overexpressing KYSE410 cells, LINC01554-transfected KYSE30 cells with compound C108 and corresponding control cells, and then observed the G3BP2 expression and tumor cell metastatic abilities. Western blot showed that the expression of G3BP2 was dramatically diminished in cells treated with compound C108 (Supplementary Fig. 6A, Fig. 6A). Due to reduced G3BP2 expression, both tumor cell migration and invasion were inhibited (Supplementary Fig. 6B, C and Fig. 6B). To further confirm the suppressive effect of compound C108 on G3BP2 expression and tumor cell metastasis, we introduced compound C108 to KYSE30 and KYSE150 cells, which had high expression of endogenous G3BP2. A reduction of G3BP2 expression, tumor cell migration, and invasion were also observed in cells treated with compound C108 (Fig. 6C, D). Moreover, in mouse model of lymph node metastasis derived from LINC01554-transfected KYSE30 cells, KYSE30, and KYSE150 cells respectively, we found compared with the vehicle control group, metastatic lymph nodes in compound C108 treatment group were smaller in size and lighter in weight (Fig. 6E–G). Metastatic lymph node sections subjected to G3BP2 and HDGF staining displayed significantly decreased expression of either G3BP2 or HDGF in the group treated with compound C108 compared with that in the control group (Fig. 6H). To further confirm that compound C108 interrupted the interaction of G3BP2 and LINC01554 by blocking RRM domain, RNA pull-down assays were conducted in KYSE30 cells treated with DMSO and compound C108. However, no significant changes of G3BP2 pulled down by LINC01554 were found (Supplementary Fig. 6D). We then wonder if compound C108 could regulate the expression of LINC01554, G3BP2 and HDGF. qRT-PCR was performed, and unexpectedly, all of them were decreased after treated compound C108 in KYSE30 and KYSE150 cells (Supplementary Fig. 6E), which indicated that compound C108 might exert the metastasis-suppressive functions through targeting various genes. Both downregulation of LINC01554 and G3BP2 would decrease the G3BP2 protein level and subsequently diminished the expression of HDGF. taken together, these results suggest that compound C108 may be used as a promising treatment for metastatic ESCC.

## Discussion

In this study, we revealed that G3BP2 was frequently upregulated in ESCC and was associated with lymph node metastasis, the depth of tumor invasion, and unfavorable outcomes in ESCC patients. Functionally, G3BP2 significantly increased the migration and invasion of ESCC cells but did not affect ESCC cell growth. The role of G3BP2 as an oncogene in ESCC was characterized. In addition to that in ESCC, we also detected the expression of G3BP2 in other types of squamous cell carcinoma, including lung squamous cell carcinoma (LUSC) and head and neck squamous cell carcinoma (HNSCC). We observed that G3BP2 was also highly expressed in both LUSC and HNSCC (Supplementary Fig. 7). These results strongly suggested that G3BP2 could be a general diagnostic biomarker and therapeutic target in squamous cell carcinoma. However, whether the functions and mechanisms of G3BP2 in LUSC and HNSCC are the same as those in ESCC deserve further investigations.

An increasing number of studies have revealed the molecular mechanism of G3BP2 in promoting cancer progression. For example, the loss of G3BP2 has been shown to promote nuclear translocation of TWIST1 with increasing matrix stiffness to drive epithelial-mesenchymal transition (EMT) tumor invasion and metastasis in breast cancer [12]. In line with the results of the above study, G3BP2 has been shown to be a treatment target in mesenchymal-like cisplatin-resistant TNBC cells, which drives S/G2 cell cycle progression to inhibit the cell response to cisplatin [13]. In addition, G3BP2 has been indicated to promote resistance to androgen deprivation therapy in prostate cancer by increasing nuclear export of P53 [14]. Besides, circFNDC3B competitively binds to miR-1178-3p, resulting in decreased expression of G3BP2, and then inhibits the downstream SRC/FAK signaling pathway to impair tumor growth and lymphatic metastasis in bladder cancer [15]. Here, we found that G3BP2 was protected by LINC01554 from ubiquitin-mediated protein degradation and stabilized HDGF mRNA transcripts to drive ESCC metastasis, which has not yet been reported during cancer progression.

So far, some mechanisms for regulating G3BP2 expression have been described. At the RNA level, G3BP2 is upregulated by androgen [14], miR-1178-3p [15], and the transcription factor FOXD1 [16]. At the protein level, the protein stability of G3BP2 is maintained by USP10 [26]. Nonetheless, how G3BP2 interacts with long noncoding RNAs has not been fully elucidated. In silico prediction suggested that G3BP2 might interact with the long noncoding RNA LINC01554. In our previous study, LINC01554 was shown to suppress tumor growth by impairing the cellular glycolytic effect in hepatocellular carcinoma [21]. However, here, our data showed that LINC01554 expression was elevated in ESCC and promoted cell migration and invasion by protecting the G3BP2 protein from proteasomal degradation. These findings suggest that LINC01554 may play various roles through distinct mechanisms in different cancers. Furthermore, according to the structural characteristics of G3BP2 and the G3BP2-binding region in LINC01554 predicted by catRAPID, we constructed plasmids with deletion of interactive regions within G3BP2 and LINC01554 and then performed a series of rescue experiments. The result revealed that G3BP2 interacted with nucleotide sequences ranging from 400 bp to 900 bp within LINC01554 through the RRM domain. As to the result that deletion of other domains of G3BP2 increased the interaction between G3BP2 and LINC01554, the conformation changes of G3BP2 after removing part of domains may explain it [27, 28]. Regarding the specific mechanisms by which LINC01554 stabilizes the G3BP2 protein, we speculate that LINC01554 may work as a scaffold for G3BP2 and deubiquitinase in stress granules since G3BP2 has been reported to interact with a deubiquitinase, USP10, in that compartment [26]. On the other hand, the binding of LINC01554 with G3BP2 may trigger the conformation changes of G3BP2, thereby evading the recognition of ubiquitinases [29, 30].

In contrast to G3BP1, which has been reported to degrade c-Myc BART, and CTNNB1 mRNA transcripts, thereby promoting cancer cell growth, metastasis and invasion [31–33], and to sequester HIV-1 by inhibiting the translation and protein packaging of viral protein [34], only the SART3 mRNA transcript has been shown to be stabilized by G3BP2, resulting in the elevated expression of pluripotent transcription factors in breast cancer initiation [11]. Thus, more RNA transcripts modulated by G3BP2 remain to be identified. Herein, the result of RNA-seq analysis highly recommended that HDGF might be regulated by G3BP2. Notably, HDGF has been shown to facilitate cell metastasis in lung cancer, Ewing sarcoma, HCC, and gastric cancer through activating EMT signaling or promoting actin cytoskeleton remodeling and cell-matrix adhesion [35–38]. We further showed that G3BP2 bound to the HDGF mRNA transcript through RRM domain and subsequently stabilized its expression in ESCC. Since HDGF and LINC01554 were both interacted with G3BP2 through RRM domain of G3BP2, whether HDGF and LINC01554 competed with each other was also investigated. Our data indicated that more HDGF transcript was enriched by anti-G3BP2 antibody after silencing LINC01554 and vice versa (Supplementary Fig. 8). Thus, in the current study, that HDGF and LINC01554 competitively interacted with G3BP2 at the same time was clarified.

To better understand the pivotal role of the LINC01554/G3BP2/HDGF signaling axis in facilitating ESCC metastasis, we designed and conducted a series of rescue experiments. The results showed that disrupting the interaction between G3BP2 and LINC01554 significantly downregulated HDGF expression and subsequently attenuated ESCC cell migration and invasion. In addition, both ectopic overexpression of LINC01554 and HDGF effectively restored the inhibitory effect of G3BP2 silencing on ESCC cell metastasis. Moreover, we found that the staining pattern of LINC01554, G3BP2, and HDGF was the same in ESCC and matched nontumor samples, which was in line with the results of the functional assays observed in vitro.

To explore whether G3BP2 is a promising therapeutic target for metastatic ESCC, a small molecule that inhibits G3BP2, compound C108, was exploited in this study. Compound C108 has been proven to inhibit tumor initiation in breast cancer in vitro and in vivo [11]. It mainly acts on the RRM domain to block the motif from binding to other molecules. However, here, we found that compound C108 inhibited G3BP2 expression not by hampering the interaction of G3BP2 and LINC01554 through RRM domain, but downregulated the RNA expression of LINC01554, G3BP2, and HDGF. Due to reduction of LINC01554, G3BP2 lost its protection from ubiquitin degradation. On the other hand, compound C108 directly declined the RNA level of G3BP2. As a molecular regulated by G3BP2, HDGF was also decreased by compound C108. But it is difficult to determine whether the reduction of HDGF is caused by decreased expression of G3BP2 or whether compound C108 directly regulates HDGF at RNA level. Hence, how compound C108 negatively regulate expression of LINC01554, G3BP2, and HDGF need to be further demonstrated in the future study. In addition, HDGF has been shown to promote tumor angiogenesis in oral cancer and HCC. In oral cancer, HDGF has been observed to induce VEGF gene expression by upregulating HIF-1α and NF-κB [39]. In HCC, HDGF antibodies obviously decrease tube formation by human umbilical vein endothelial cells [40]. Since angiogenesis is closely related to tumor metastasis, it can be a novel therapeutic strategy to combine compound C108 with antiangiogenic agents or antibodies against HDGF in the treatment of metastatic ESCC, which may achieve better treatment effects.

In conclusion, our findings (Fig. 6I) identify a novel regulatory signaling axis, LINC01554/G3BP2/HDGF, that facilitates ESCC metastasis, which may provide rational strategies for developing potential biomarkers and therapeutic targets for ESCC.

## Materials and methods

The materials and methods in detail were provided in the supplementary information file. The primer sequences are listed in Supplementary Table 2. The sequences of the shRNAs and siRNAs are listed in Supplementary Table 3.

## Supplementary information

Supplementary information file

## Data Availability

All data generated/analyzed during this study are available from the corresponding author on reasonable request.
