# Supplementary material for: G3BP2 regulated by the lncRNA LINC01554 facilitates esophageal squamous cell carcinoma metastasis through stabilizing HDGF transcript
Source: Oncogene. 2021 Nov 15;41(4):515–26. doi: 10.1038/s41388-021-02073-0 (PMC8782723; doi:10.1038/s41388-021-02073-0)
Supplement: Supplementary file 1 — Supplementary information file [file 41388_2021_2073_MOESM1_ESM.docx]

**Supplementary information file**

**G3BP2 regulated by the lncRNA LINC01554 facilitates esophageal squamous cell carcinoma metastasis through stabilizing HDGF transcript**

**Zheng et al.**

**Supplementary materials and methods**

**Supplementary figures**

**Supplementary Figure 1:** Protein expression of G3BP2 was determined by Western blot in 8 paired fresh ESCC tissues and corresponding nontumor samples.

**Supplementary Figure 2:** LINC01554 regulates G3BP2 not at the RNA level but at the protein level mediated by proteasome system through binding to RRM domain of G3BP2.

**Supplementary Figure 3:** G3BP2 did not affect ESCC cell growth.

**Supplementary Figure 4:** G3BP2 stabilized HDGF mRNA transcript though RRM domain and HDGF was highly expressed in ESCC.

**Supplementary Figure 5:** LINC01554/G3BP2/HDGF signaling axis facilitates ESCC cell metastasis.

**Supplementary Figure 6:** Compound C108 exerted the metastasis-suppressive function not through blocking RRM domain of G3BP2 but downregulating the expression of LINC01554, G3BP2 and HDGF.

**Supplementary Figure 7:** Expression of G3BP2 was evaluated by IHC staining in LUSC and HNSCC tissue microarray.

**Supplementary Figure 8:** HDGF and LINC01554 competitively interacted with G3BP2.

**Supplementary Tables**

**Supplementary Table 1.** Clinicophathologic correlation of G3BP2 expression in ESCC.

**Supplementary Table 2.** Sequences of primers used in RT-qPCR assay.

**Supplementary Table 3.** Sequences of shRNAs and siRNAs used in this study.

**Supplementary materials and methods**

**Clinical samples and cell lines**

Thirty pairs of fresh primary ESCC and corresponding nontumor tissues were collected immediately after surgical resection at Sun Yat-sen University Cancer Center (SYSUCC, Guangzhou, China). A tissue microarray (TMA) containing 183 ESCC samples and 93 matched nontumor tissue samples were constructed from the paraffin-embedded tissues collected from SYSUCC. The use of all the clinical samples in this study was authorized by the Committees for Ethical Review of Research at Sun Yat-sen University Cancer Center. Six ESCC cell lines (KYSE30, KYSE180, KYSE510, KYSE140, KYSE150 and KYSE410) were obtained from DSMZ (Braunschweig, Germany), the German Resource Centre for Biological Material. The immortalized NE1 esophageal epithelial cell line was purchased from the American Type Culture Collection (ATCC, Manassas, Virginia, USA). NE1 cells were cultured in specific medium containing keratinocyte-SFM (Invitrogen, Carlsbad, CA) and EpiLife (Thermo Scientific, Rockford, IL), and other cell lines were cultured in RPMI 1640 medium (GibcoBRL, Grand Island, NY) supplemented with 10% fetal bovine serum (FBS, GibcoBRL, Grand Island, NY). All the cell lines were authenticated by STR profiling and tested for mycoplasma contamination (MycoAlert, Lonza).

**Immunohistochemistry (IHC) staining**

IHC staining was performed according to a standard streptavidin-biotin-peroxidase complex method [1,2]. Parafﬁn-embedded, formalin-ﬁxed sections were dewaxed with xylene and blocked in 3% hydrogen peroxide solution. Antigen retrieval was performed by pressure cooking the samples in citrate buffer (pH 6.0) for 3 min. The slides were incubated overnight with primary antibodies (anti-G3BP2: 1:3000, #HPA018304, Sigma-Aldrich, Saint Louis, MO; anti-HDGF: 1:4000, #11344-1-AP, Proteintech, IL, USA) at 4 °C. Staining was visualized by sequential incubations of the slides in an Envision detection system (Dako, Glostrup, Denmark), and nuclei were counterstained with Meyer’s hematoxylin. Immunohistochemical staining of G3BP2 was evaluated by two pathologists blinded to the patients’ characteristics. The proportion of immune-positive cells was scored on a scale of 0–4 (0%, 1–25%, 26–50%, 51–75%, and 76–100%). The staining intensity was scored as negative (0), weak (1), medium (2), and strong (3). The score was calculated by multiplying the proportion of immune-positive cells with the intensity score, and the median was selected as the cut-off value to determine upregulation (score ≥ 6) and downregulation (score < 6) of G3BP2.

**RNA isolation and** **real-time quantitative PCR (RT-qPCR)**

Total RNA was extracted by TRIzol (Invitrogen, Carlsbad, CA) following the manufacturer’s instructions. Reverse transcription was subsequently conducted using the HiScript II Q RT SuperMix Kit (Vazyme Biotech, Nanjing, China). RT-qPCR was carried out using the SYBR Green method (Roche, Basel, Switzerland) on a Roche LightCycler® 480 PCR system (Roche, Basel, Switzerland). GAPDH was used as an internal control. The primer sequences are listed in Supplementary Table 2.

**Plasmid construction**

pcDNA3.1-G3BP2, pcDNA3.1-LINC01554, pcDNA3.1-HDGF and Psi-LVRU6GP-shRNAs targeting G3BP2 and the corresponding control plasmids were purchased from GeneCopoeia (Rockville, MD). There two shRNAs (shRNA#1 and shRNA#2) constructed to silence G3BP2. Plasmids with shRNAs targeting G3BP2 and a short hairpin negative control sequence were transfected into 293FT cells using lentiviral packaging mix (Invitrogen, Carlsbad, CA), respectively. Western blot was used to detect the knockdown efficiency of G3BP2 in KYSE30 and KYSE150 cells and showed the greater decrease in G3BP2 after transfection with shRNA#1 (Data not shown). Thus，shRNA#1 was used for the subsequent experiments. siRNAs targeting LINC01554 and HDGF were purchased from RiboBio (Guangzhou, China) and Genepharma (Shanghai, China), respectively. The sequences of the shRNAs and siRNAs are listed in Supplementary Table 3.

***In vitro* metastasis assay**

For the wound healing assay, cells were seeded in six-well plates and wounded with a 200 μL pipette tip when the cell monolayers were confluent. Images were captured 24 h after scratching under an inverted light microscope (Olympus, Lake Success, NY). The migration assays and invasion assays were conducted using transwell chambers (pore size 8 μm) with or without Matrigel membranes (Corning, NY, USA). Briefly, cells were digested with trypsin and resuspended in 200 μL of serum-free RPMI 1640 medium and were then added to the upper chamber, while 600 μL of complete RPMI 1640 medium was added to the lower chamber. After 12 h (for KYSE30 cells), 24 h (for KYSE410 cells), and 30 h (for KYSE150 and KYSE510 cells), migrating or invading cells attached to the lower surface of the membrane were fixed with a paraformaldehyde solution, stained with 0.1% crystal violet and counted under an objective lens.

***In vivo* metastatic assay**

The *in vivo* metastatic assay was approved by the SYSUCC Animal Care and Use Committee (IACUC) following its guidelines. Both lymph node and lung metastatic models were established in 5-week-old BALB/c nude male mice to evaluate the metastatic effect of G3BP2. A total of 5×10^5^ cells were injected into the footpad of lymph node metastatic model mice (n = 5 for KYSE30 cells and n = 6 for KYSE150 cells), while 1×10^6^ cells were intravenously administered via the tail vein to lung metastatic model mice (n = 6 for KYSE150 cells). After 4 to 5 weeks, the mice were sacrificed, and the inguinal lymph nodes or lungs were isolated. All the samples were embedded in paraffin and stained with H&E.

**Cell growth assay and foci formation assay**

In order to investigate cell growth, 1×10^3^ cells (shG3BP2#1, shG3BP2#2 and shCtrl derived from KYSE150 cells) were seeded in 96-well plates and the cell proliferation rate was examined using a Cell Counting Kit-8 kit (Dojindo Molecular Technologies, Inc., Kumamoto, Japan), according to the manufacturer's protocol. For foci formation assays, 1×10^3^ cells were seeded in 6-well plates, and cultured for a week. Colonies comprised of >50 cells stained with 1% crystal violet and counted.

**LncRNA fluorescence *in situ* hybridization (FISH) and immunofluorescence staining**

RNA FISH was performed on ESCC tumor and paired nontumor tissues using an RNA fluorescence *in situ* hybridization kit (Exonbio Lab, Guangzhou, China) as previously described [3]. The colocalization of G3BP2 (1:100, #16276-1-AP, Proteintech, IL, USA) and LINC01554 in ESCC cells was determined using RNA FISH and immunofluorescence staining. Images were captured by confocal microscopy (Olympus FV1000, Tokyo, Japan).

**RNA immunoprecipitation assay**

RNA immunoprecipitation (RIP) assays were conducted using 5 μg of anti-G3BP2 antibody (#16276-1-AP, Proteintech, IL) and an RNA-binding protein immunoprecipitation kit (Millipore, Burlington, MA) according to the manufacturer’s instructions. The enrichment of LINC01554 and HDGF was determined by RT-qPCR.

**RNA pull-down assay**

RNA pull-down assays were performed using a Pierce magnetic RNA-protein pull-down kit (Thermo Scientific, Rockford, IL) according to the manufacturer’s instructions. Western blot was used to detect the target protein.

**Determination of mRNA half-life**

Cells were seeded in six-well plates and treated with actinomycin D (5 µg/mL, TargetMol, China) the next day for different durations. Total RNA was then extracted and subjected to RT-qPCR. Setting the values at time 0 without actinomycin D as 100%, a mRNA decay curve was constructed. The time point corresponding to 50% remaining mRNA was considered the mRNA half-life. The experiments were repeated three times.

**Cycloheximide chase assay**

To assess the protein stability, cycloheximide (CHX) (MP Biomedicals, Santa Ana, CA) was introduced to the cell culture medium at a final concentration of 10 μM. Total protein was extracted at the indicated time points and determined by Western blot.

***In vitro* ubiquitination assay**

Immunoprecipitation was performed using a Pierce direct magnetic IP kit (Thermo Scientific, Rockford, IL) according to the manufacturer’s instructions. Briefly, cell lysates were collected after the introduction of MG132 (10 μM, Selleck Chemicals, Houston, TX) for 10 h, incubated overnight with 5 μg of anti-G3BP2 antibody (#16276-1-AP, Proteintech, IL) at 4 °C, eluted with 50 μL of lysis buffer and denatured with SDS sample buffer. The elution was analyzed by Western blot with an anti-ubiquitin antibody (1:1000, #3936, Cell Signaling Technology, Danvers, MA).

**Antibodies and Western blot assay**

Western blot assays were performed according to the standard protocol. Antibodies against the following proteins were used: anti-G3BP2 (1:1000, #16276-1-AP, Proteintech, IL), anti-HDGF (1:1000, #11344-1-AP, Proteintech, IL), and anti-GAPDH (1:3000, #AP7873a, Abgent, San Diego, CA).

**RNA sequencing analyses**

Total RNA was extracted from G3BP2 knockdown cells by shRNA and control cells derived from KYSE30 and KYSE150 cells. RNA sequencing was conducted by Novogene (Beijing, China). Both RNA purity and integrity were examined before transcriptome sequencing. All data analyses were based on high-quality clean data. Differential expression analysis was performed using the DESeq2 R package. The resulting *P* values were adjusted using Benjamini and Hochberg’s approach for controlling the false discovery rate. Genes with an adjusted P value <0.05 identified by DESeq2 were considered to be differentially expressed.

**Treatment with the G3BP2 inhibitor**

G3BP2- and LINC01554-transfected cells, as well as two cells with high endogenous expression of G3BP2, KYSE30 and KYSE150 cells were treated with the G3BP2 inhibitor compound C108 (4 μM, Cayman Chemical, Michigan) or DMSO for 24 h to examine its effects on cell migration and invasion *in vitro*. For the *in vivo* assay, LINC01554-transfected KYSE30 cells, KYSE30 and KYSE150 cells were treated with compound C108 or DMSO for 24 h before injection into the footpads of mice (n = 6). Each group consisted of six 5-week-old BALB/c male nude mice. After 4 to 5 weeks, all the mice were sacrificed, and the inguinal lymph nodes were weighed and dissected.

**Statistical analysis**

Data analyses were performed using SPSS version 22.0 (Chicago, IL) and GraphPad Prism 7.0. A paired two-tailed Student’s *t*-test was used for the analysis of G3BP2, LINC01554 mRNA levels and IHC staining score of G3BP2 in ESCC tumor and corresponding nontumor specimens. Kaplan–Meier plots and log-rank tests were used for overall survival analysis. Pearson’s chi-square test was applied to analyze clinical correlations between G3BP2 and clinicopathologic parameters. Other significant differences between two groups were analyzed by unpaired *t*-test. All results are presented as the mean ± SD, and results with *P* < 0.05 were considered to be statistically significant.

**References**

1. Li L, Zheng YL, Jiang C, Fang S, Zeng TT, Zhu YH, et al. HN1L-mediated transcriptional axis AP-2γ/METTL13/TCF3-ZEB1 drives tumor growth and metastasis in hepatocellular carcinoma. Cell Death Differ. 2019;26:2268-83.
2. Zhang B, Zhang Z, Li L, Qin YR, Liu H, Jiang C, et al. TSPAN15 interacts with BTRC to promote oesophageal squamous cell carcinoma metastasis via activating NF-κB signaling. Nat Commun. 2018;9:1423.
3. Zheng YL, Li L, Jia YX, Zhang BZ, Li JC, Zhu YH, et al. LINC01554-mediated glucose metabolism reprogramming suppresses tumorigenicity in hepatocellular carcinoma via downregulating pkm2 expression and inhibiting akt/mtor signaling pathway. Theranostics. 2019;9:796-810.

**Supplementary figures**


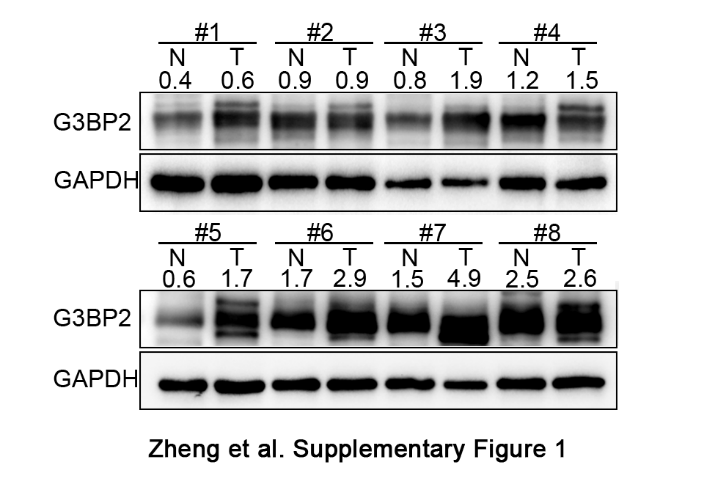


**Supplementary Figure 1** **Protein expression of G3BP2 was determined by Western blot in 8 paired fresh ESCC tissues and corresponding nontumor samples.** GAPDH serves as internal control.


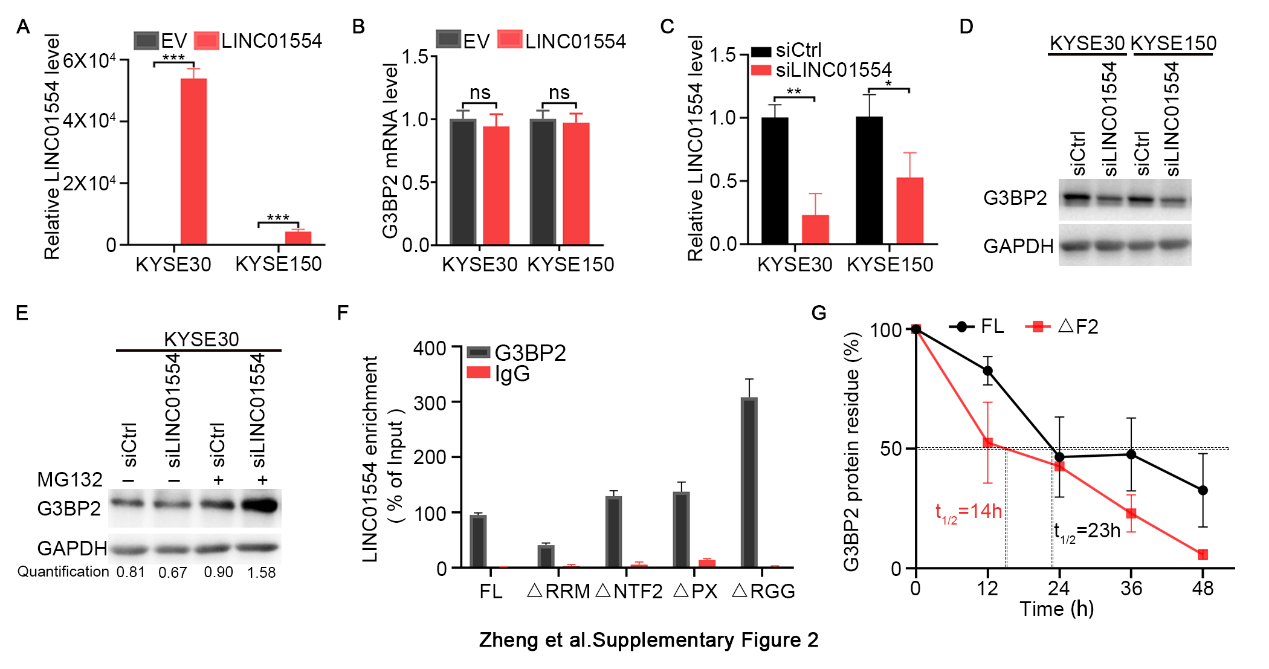


**Supplementary Figure 2** **LINC01554 regulates G3BP2 not at the RNA level but at the protein level mediated by proteasome system through binding to RRM domain of G3BP2.** (A) qRT-PCR examined the overexpressing efficiency of LINC01554 in KYSE30 and KYSE150 cells. GAPDH served as internal control. (B) qRT-PCR detected mRNA expression level of G3BP2 in LINC01554 transfected KYSE30 and KYSE150 cells, normalized by empty control cells. GAPDH served as internal control. ns, nonsignificant. (C) qRT-PCR evaluated the silencing efficiency of LINC01554 in KYSE30 and KYSE150 cells. GAPDH served as internal control. (D) Western blot analysis showed the reduction of G3BP2 protein expression in LINC01554-knockdown KYSE30 and KYSE150 cells. (E) Western blot analysis revealed that the decreasing of G3BP2 induced by silencing LINC01554 was attenuated by MG132. (F) RIP assays were performed in cells transfected with a series of truncated G3BP2 domain constructs. IgG antibody served as a control for each RIP assay. FL, full-length G3BP2; ΔRRM, truncation of the RRM domain in G3BP2; ΔNTF2, truncation of the nuclear transport factor 2-like domain in G3BP2; ΔPX, truncation of the acidic and proline-rich regions in G3BP2; ΔRGG, truncation of the arginine and glycine rich box domain in G3BP2. (G) Quantification of Western blot bands in Fig.2R. The data represent the mean ± SD. **P* < 0.05, ***P* < 0.01, ****P* < 0.001.


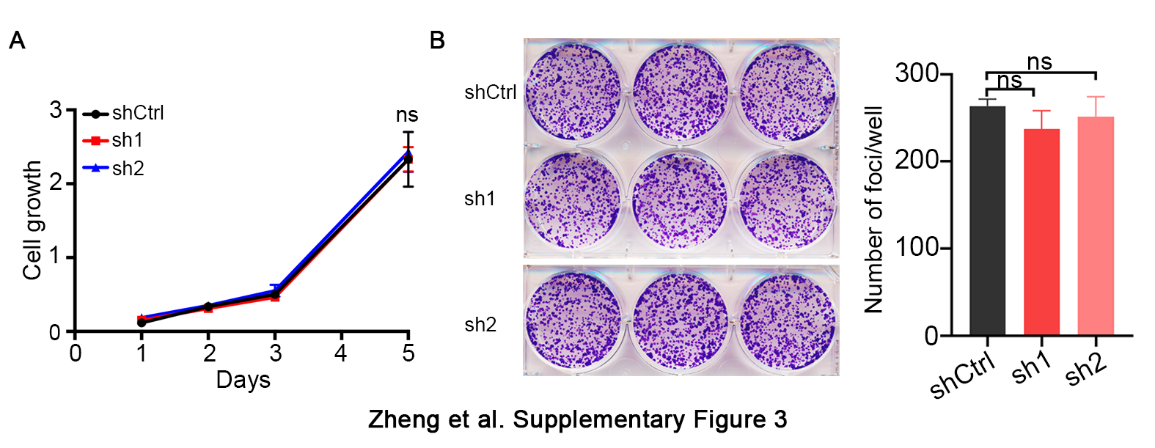


**Supplementary Figure 3** **G3BP2 did not affect ESCC cell growth.** (A) CCK8 proliferation assay revealed that there were no differences in cell proliferation between KYSE150 cells with or without G3BP2 silencing by shRNAs. (B) Foci formation assay showed that there was no difference in the number of foci formed per well between KYSE510 cells with G3BP2 silencing and control cells. The data represent the mean ± SD. ns, nonsignificant.


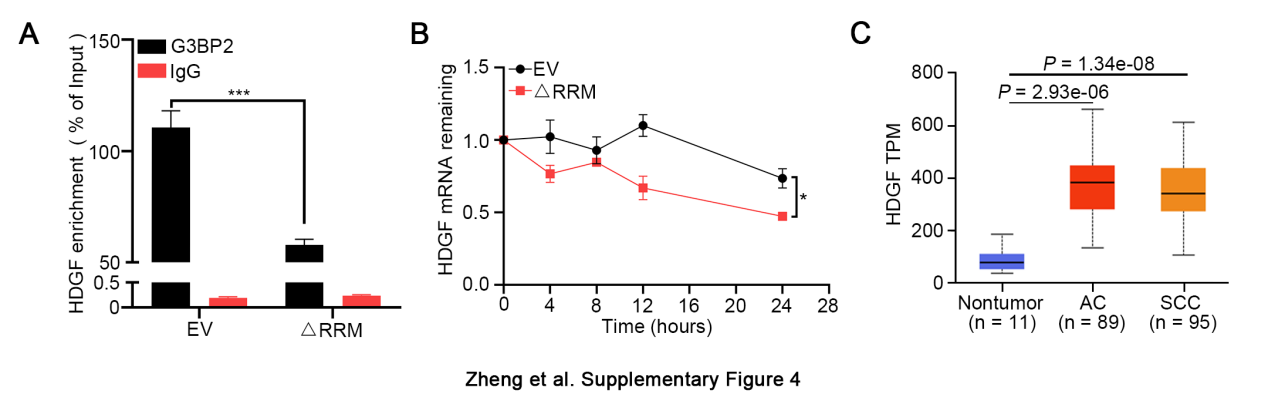


**Supplementary Figure 4** **G3BP2 stabilized HDGF mRNA transcript though RRM domain and HDGF was highly expressed in ESCC.** (A) RIP assay showed that the interaction between G3BP2 and HDGF transcript was weakened after deletion of the RRM domain of G3BP2 in KYSE30 cells. IgG antibody served as a control for the RIP assay. ΔRRM-G3BP2, truncation of the RRM domain in G3BP2. (B) The half-life of HDGF mRNA transcript was significantly shortened in KYSE30 cells with truncation of the RRM domain of G3BP2. The data represent the mean ± SD from three independent experiments. **P* < 0.05. (C) Big data mining (http://ualcan.path.uab.edu) showed that HDGF was upregulated in ESCC.


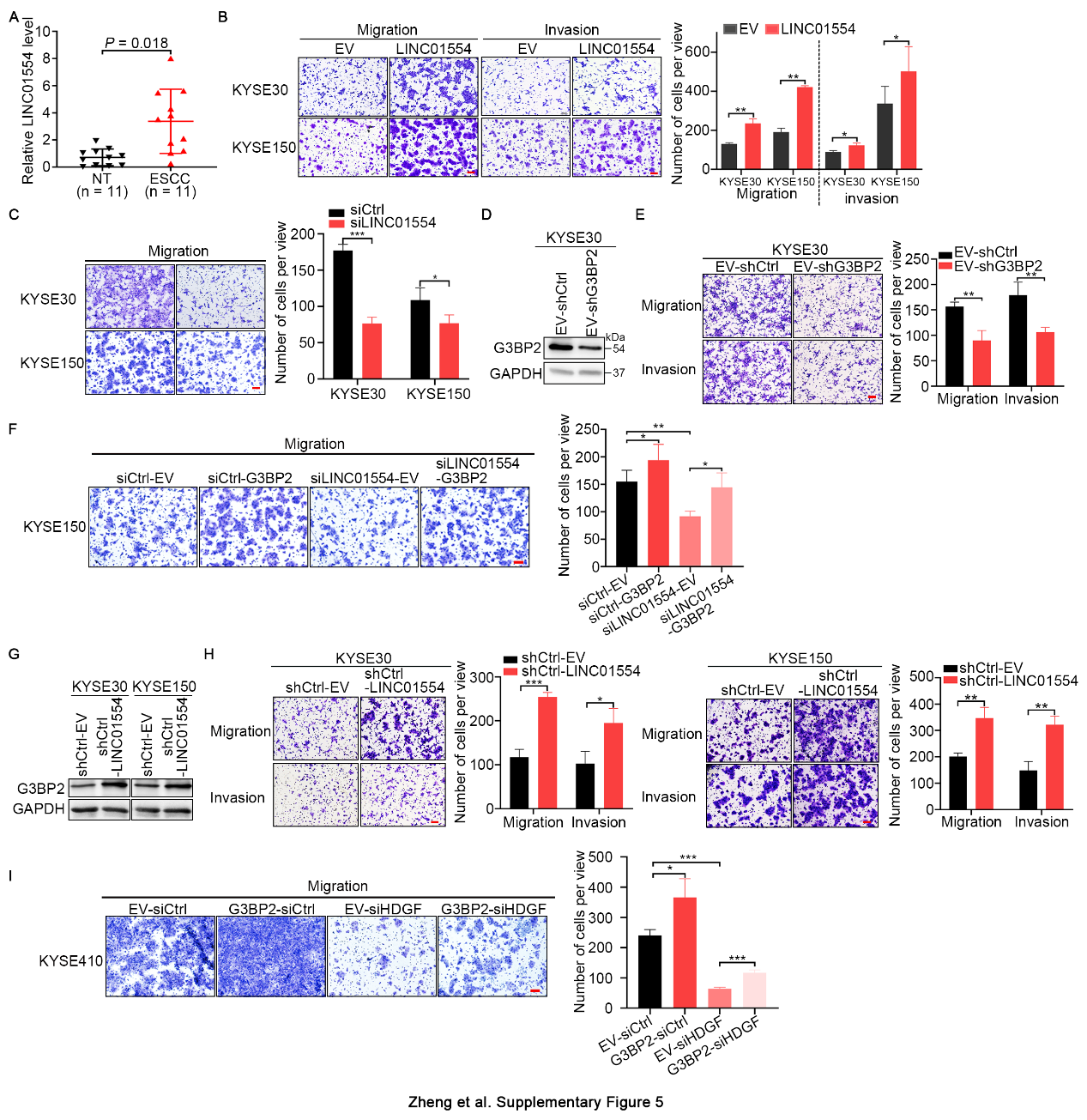


**Supplementary Figure 5 LINC01554/G3BP2/HDGF signaling axis facilitates ESCC cell metastasis.** (A) Expression of LINC01554 was determined by qRT-PCR in 11 paired ESCC and matched nontumor samples from SYSUCC. GAPDH served as an internal control. NT, adjacent nontumor tissue. (B) Representative images from transwell assays showed that cell migration and invasion ability were bolstered in LINC01554-transfected KYSE30 and KYSE150 cells. The statistical analysis results are presented in the right panel. (C) Representative images from transwell assays showed that silencing LINC01554 inhibited migration of ESCC cells. The statistical analysis results are presented in the right panel. (D) The knockdown efficiency of G3BP2 in empty vector-KYSE30 cells were determined by Western blot. (E) Representative images from transwell assays indicated that silencing G3BP2 impaired migratory and invasive capabilities of empty vector-KYSE30 cells. The statistical analysis results are presented in the right panel. (F) Representative images from transwell assays revealed that overexpressing G3BP2 abolished the migration-suppressive effect of silencing LINC01554 in KYSE150 cells. The statistical analysis results are presented in the right panel. (G) Western blot analysis showed that the G3BP2 expression was increased after transfecting LINC01554 in shCtrl-KYSE30 and -KYSE150 cells. (H) Representative images from transwell assays indicated that LINC01554 promoted migration and invasion of shCtrl-KYSE30 and -KYSE150 cells. The statistical analysis results are presented in the right panel. (I) Representative images from transwell assays showed that the migration-promoting role of G3BP2 was attenuated by knocking down HDGF in KYSE410 cells. The statistical analysis results are presented in the right panel. Scale bar, 50 μm. The data are expressed as the mean ± SD from triplicate experiments. **P* < 0.05, ***P* < 0.01, ****P* < 0.001.


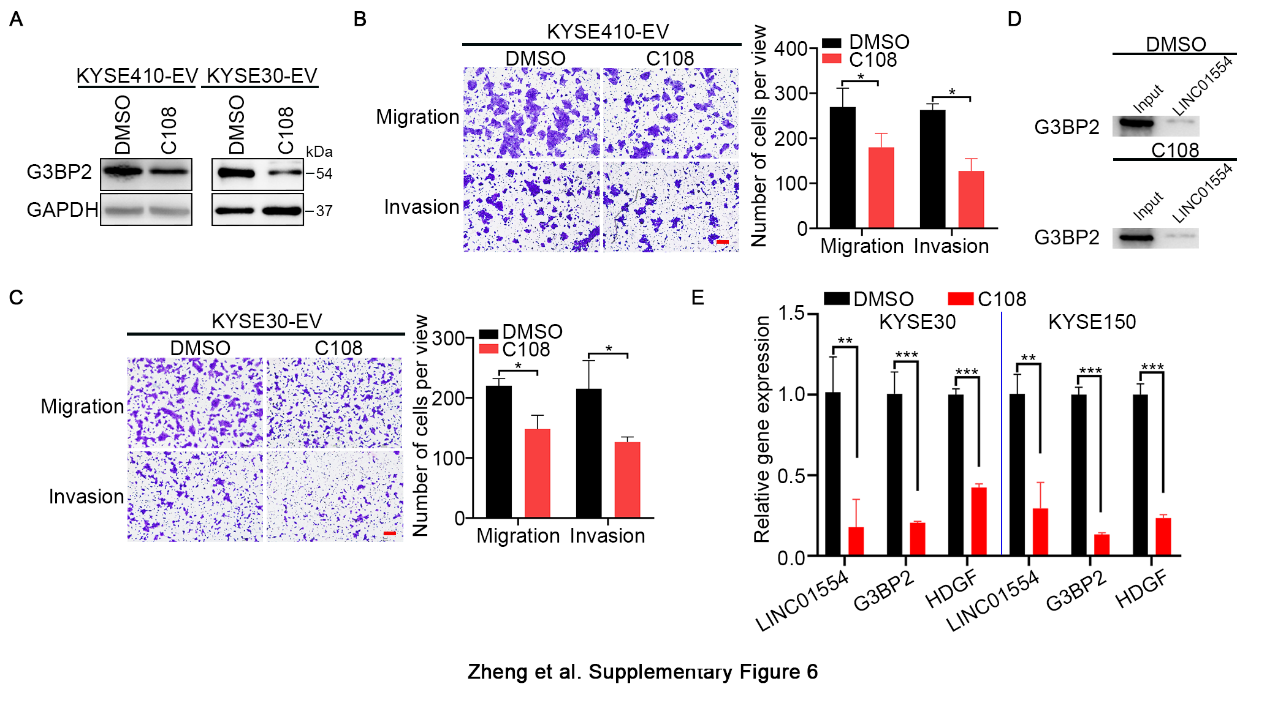


**Supplementary Figure 6 Compound C108 exerted the metastasis-suppressive function not through blocking RRM domain of G3BP2 but downregulating the expression of LINC01554, G3BP2 and HDGF.** (A) Western blot analysis showed that the G3BP2 expression was decreased after being treated with compound C108 in empty vetor-KYSE410 and -KYSE30 cells. (B, C) Representative images from transwell assays indicated that compound C108 inhibited migration and invasion of empty vetor-KYSE410 and -KYSE30 cells. The statistical analysis results are presented in the right panel. (D) Western blot analysis with anti-G3BP2 in KYSE30 cells treated with and without compound C108 followed by RNA pull-down assay with LINC01554 probes. (E) Expression of LINC01554, G3BP2 and HDGF was determined by qRT-PCR in KYSE30 and KYSE150 cells treated with and without compound C108. Scale bar, 50 μm. The data are expressed as the mean ± SD from triplicate experiments. **P* < 0.05, ***P* < 0.01, ****P* < 0.001.


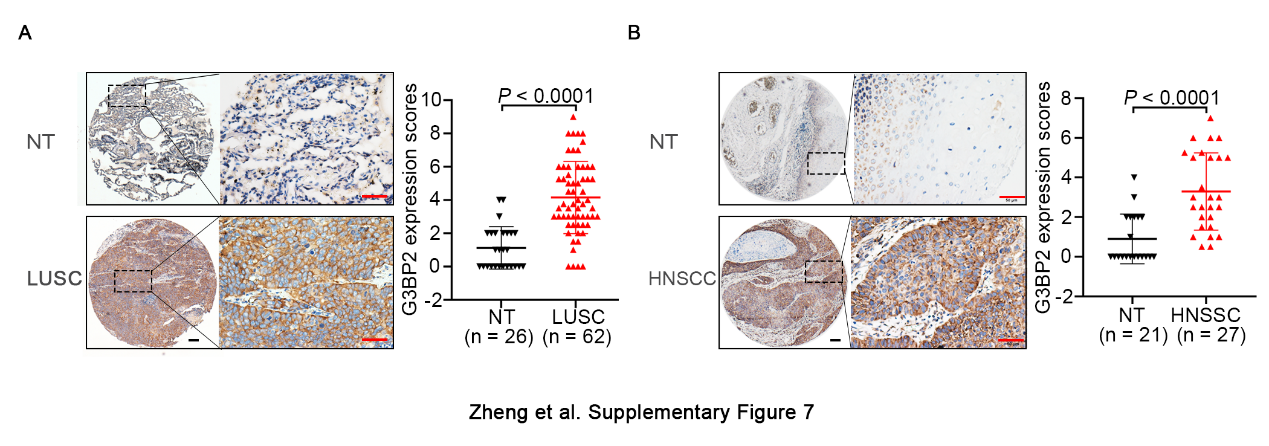


**Supplementary Figure 7** **Expression of G3BP2 was evaluated by IHC staining in LUSC and HNSCC tissue microarray.** (A) Representative images and G3BP2 staining score in nontumor tissue and LUSC tissue. Scale bar, left: 100 μm, right: 50 μm. (B) Representative images and score of G3BP2 staining in nontumor tissue and HNSCC tissue. Scale bar, left: 100 μm, right: 50 μm. The data are represented as the mean ± SD. NT, adjacent nontumor tissue.


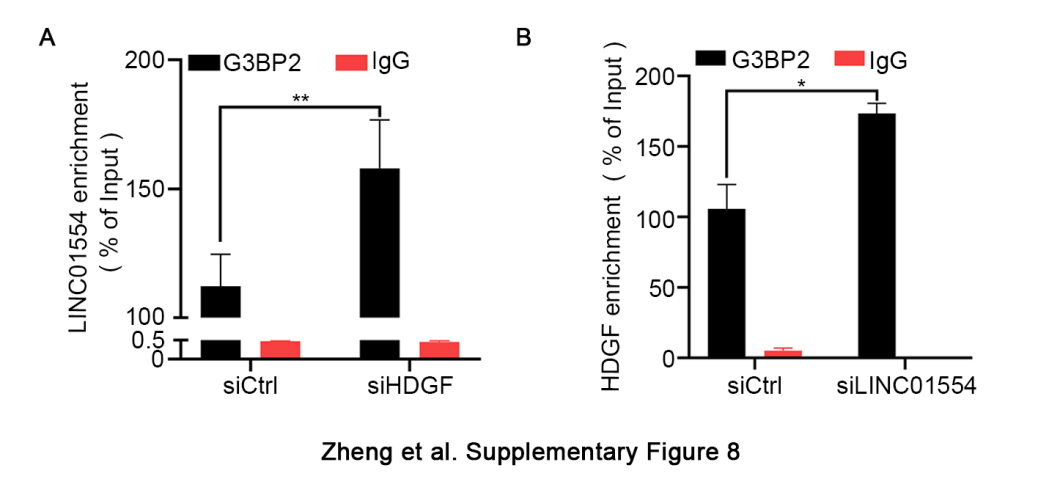


**Supplementary Figure 8 HDGF and LINC01554 competitively interacted with G3BP2.** (A) The enrichment of LINC01554 transcript was detected by qRT-PCR followed by RIP assay in KYSE30 cells with HDGF silencing. (B) The enrichment of HDGF transcript was detected by qRT-PCR followed by RIP assay in KYSE30 cells with LINC01554silencing. IgG antibody served as a control for the RIP assay. The data are expressed as the mean ± SD from triplicate experiments. **P* < 0.05, ***P* < 0.01.

**Supplementary Table 1.** Clinicophathologic correlation of G3BP2 expression in ESCC

| Features | Total | G3BP2 expression | | *P* |
| --- | --- | --- | --- | --- |
|  |  | Low (N = 43) | High (N = 50) |  |
| Gender |  |  |  |  |
| Male | 73 | 33 (45.2%) | 40 (54.8%) | 0.703 |
| Female | 20 | 10 (50%) | 10 (50%) |  |
| Age (years) |  |  |  |  |
| ≤60 | 43 | 23 (53.3%) | 20 (46.5%) | 0.304 |
| >60 | 50 | 32 (64.0%) | 18 (36.0%) |  |
| Differentiation |  |  |  |  |
| High | 29 | 16 (37.2%) | 13 (26.0%) | 0.342 |
| Moderate | 41 | 19 (44.2%) | 22 (44.0%) |  |
| Low | 23 | 8 (18.6%) | 15 (30.0%) |  |
| Lymph node metastasis |  |  |  |  |
| No | 47 | 28 (59.6%) | 19 (40.4%) | **0.009^*^** |
| Yes | 46 | 15 (32.6%) | 31 (67.4%) |  |
| Distant metastasis |  |  |  |  |
| No | 87 | 40 (46..0%) | 47 (54.0%) | 0.848 |
| Yes | 6 | 3 (50.0%) | 3 (50.0%) |  |
| Invasive depth |  |  |  |  |
| Submucosa | 17 | 12 (70.6%) | 5 (29.4%) | **<0.001^*^** |
| Circular muscularis | 16 | 11 (68.8%) | 5 (31.2%) |  |
| Longitudinal muscularis | 13 | 9 (69.2%) | 4 (30.8%) |  |
| Adventitia | 47 | 11 (23.4%) | 36 (76.6%) |  |
| Tumor size (cm) |  |  |  |  |
| ≤7.5 | 34 | 19 (55.9%) | 15 (44.1%) | 0.157 |
| >7.5 | 59 | 24 (40.7%) | 35 (53.8%) |  |
| Tumor staging |  |  |  |  |
| I-II | 51 | 24 (47.1%) | 27 (52.9%) | 0.861 |
| III-IV | 42 | 19 (45.2%) | 23 (54.8%) |  |

**Supplementary Table 2.** Sequences of primers used in RT-qPCR assay

| Target | Sequence |
| --- | --- |
| GAPDH | Forward: 5'-GTCTCCTCTGACTTCAACAGCG-3'  Reverse: 5'-ACCACCCTGTTGCTGTAGCCAA-3' |
| G3BP2 | Forward: 5'-CCTCGTGTGCGTGAACAAC-3'  Reverse: 5'-CATGTGGCAAGTTACCAACAAAA-3' |
| LINC01554 | Forward: 5'-GAGGGCAAAAGACTGCAAGC-3'  Reverse: 5'-CTCATCAACCGACCTCCCTG-3' |
| HDGF | Forward: 5'-AACAACCCTACTGTCAAGGCT-3'  Reverse: 5'-TCTTCAACGCTCCTTTCTCGT-3' |

**Supplementary Table 3.** Sequences of shRNAs and siRNAs used in this study

| shRNA/siRNA | Sequence |
| --- | --- |
| shG3BP2#1 | CGGGAGTTTGTGAGGCAATAT |
| shG3BP2#2 | GACTCTGACAACCGTAGAATA |
| siLINC01554#1 | GCAAGCAGAATATCCCTTT |
| siHDGF#2 | CUACCA AGG AAGAUGCUGA |
